# Supplementary material for: Where’s Whaledo: A software toolkit for array localization of animal vocalizations
Source: PLoS Comput Biol. 2024 May 20;20(5):e1011456. doi: 10.1371/journal.pcbi.1011456 (PMC11142720; doi:10.1371/journal.pcbi.1011456)
Supplement: S1 Text — (PDF) [file pcbi.1011456.s001.pdf]

Derivation of TDOA uncertainties σ_{sml} and σ_{lrg}

Eric R. Snyder[1*], Alba Solsona-Berga [1], Simone Baumann-Pickering[1], Kait E. Frasier[1], Sean M. Wiggins[1], John A. Hildebrand[1]

1 Scripps Institution of Oceanography, University of California San Diego, La Jolla, CA, USA

* e5snyder@ucsd.edu

Derivation of Uncertainty

The standard deviations used in the Maximum Likelihood equations were assumed independent, and therefore the propagation of uncertainty for a function $f(x_1, x_2, ...)$ can be approximated as:

$$\sigma_f = \sqrt{\left(\frac{\partial f}{\partial x_1}\right)^2 \sigma_{x_1}^2 + \left(\frac{\partial f}{\partial x_2}\right)^2 \sigma_{x_2}^2 + ...} \tag{1}$$

Applying this to the case where $f = x_1 x_2$ or $f = \frac{x_1}{x_2}$ gives:

$$\sigma_f = |f|\sqrt{\left(\frac{\sigma_{x_1}}{x_1}\right)^2 + \left(\frac{\sigma_{x_2}}{x_2}\right)^2}. \tag{2}$$

Two standard deviations were used: the uncertainty in the large aperture TDOA (σ_{lrg}), and the small aperture TDOA (σ_{sml}). The errors are due to differences between measured TDOAs and the TDOAs calculated in the model:

The small-aperture error equation is

$$\epsilon = |\text{TDOA}_{\text{calc}} - \text{TDOA}_{\text{meas}}|.$$

The calculated large-aperture TDOA is given by:

$$\text{TDOA}_{\text{lrg,calc}} = \frac{R_i - R_j}{c}. \tag{3}$$

where R_i is the distance between the ith instrument and the whale location:

$$R_i = \sqrt{(w_x - h_{i,x})^2 + (w_z - h_{i,z})^2 + (w_z - h_{i,z})^2}. \tag{4}$$

For the small-aperture TDOA, we use the plane-wave approximation (as mentioned in the paper):

$$\text{TDOA}(k,l)_{\text{sml,calc}} = \frac{\vec{H}_{k,l} \cdot \vec{s}}{c}, \tag{5}$$

where $\vec{H}_{k,l}$ is the vector between the kth and l^{th} hydrophone elements in the array, and $\vec{s}$ is the unit vector representing the direction from the source to the array ($\vec{s} = \frac{\vec{w} - \vec{h}_i}{R_i}$).

Small-aperture TDOA uncertainy, $\sigma_{\mathbf{sml}}$

Sources of uncertainty on the small-aperture array are:

- σ_{h_i} – uncertainty in the ith instrument location,
- σ_{ray} – uncertainty in the sound speed with depth resulting in ray bending,
- $\sigma_{H_{k,l}}$ – uncertainty in the vector $\vec{H}_{k,l}$ connecting the nth and m^{th} hydrophone elements within a small aperture array,
- σ_c – uncertainty due to changes in sound speed at the instrument depth over time, and
- σ_{xcorr} – uncertainty due to the precision of cross-correlation in determining the TDOA.

Estimating σ_{h_i} and $\sigma_{\mathbf{ray}}$

σ_{h_i} and σ_{ray} both contribute to the uncertainty in $\vec{s}$. h_i is related to $\vec{s}$ by $\vec{s} = \frac{\vec{w}-\vec{h}_i}{R_i}$. Using Eq 1, we calculate the error on $\vec{s}$ as follows:

$$\sigma_{\vec{s}} = \sqrt{\left(\frac{\partial s_x}{\partial h_{i,x}}\right)^2 \sigma_{h_i}^2 + \left(\frac{\partial s_y}{\partial h_{i,y}}\right)^2 \sigma_{h_i}^2 + \left(\frac{\partial s_z}{\partial h_{i,z}}\right)^2 \sigma_{h_i}^2}. \tag{6}$$

Deriving partial derivatives gives:

$$\frac{\partial s_x}{\partial h_{i,x}} = \frac{-(w_y - h_{i,y})^2 - (w_z - h_{i,z})^2}{R_i^3} = -\frac{1}{R_i}\left(s_y^2 + s_z^2\right),$$
$$\frac{\partial s_y}{\partial h_{i,y}} = \frac{-(w_x - h_{i,x})^2 - (w_z - h_{i,z})^2}{R_i^3} = -\frac{1}{R_i}\left(s_x^2 + s_z^2\right),$$
$$\frac{\partial s_z}{\partial h_{i,z}} = \frac{-(w_x - h_{i,x})^2 - (w_y - h_{i,y})^2}{R_i^3} = -\frac{1}{R_i}\left(s_x^2 + s_y^2\right).$$

Plugging this in to Eq. 6 and rearranging leads to:

$$\sigma_{\vec{s}} = \sqrt{\left(\frac{2\sigma_{h_i}}{R_i}\right)^2 \left(s_x^2 + s_y^2 + s_z^2\right)^2} = \frac{2\sigma_{h_i}}{R_i}.$$

This derivation causes the uncertainty in $\vec{s}$ to approach infinity as the range between the source and receiver array approaches zero. In these situations, however, the plane-wave approximation also breaks down since the range to the array would be smaller than the distance between array elements. The value of $\frac{2\sigma_{hi}}{R_i}$ diminishes quickly with range. When localizing with the maximum likelihood equations, we need a single value for $sigma_{\mathrm{sml}}$ for all grid locations and for both arrays. If the selected value of R_i is too low we disproportionately penalize the small-aperture TDOA by overestimating the uncertainty; however, if it is too high, we risk underestimating the actual uncertainty. After analyzing many tracks, it became apparent that a vast majority of the detections came from hundreds of meters away, and on occasions when a whale passed close to an array it spent a relatively short portion of its time within 100 m. Thus, a value of $R_i = 100$ was selected for this analysis. This approximation may need to be reconsidered for configurations where animals are more closely approaching the receivers.

Incorporating the uncertainty due to ray bending yields the equation:

$$\sigma_{\vec{s}} \approx \sqrt{\frac{1}{100^2}\sigma_{h_i}^2 + \sigma_{\text{ray}}^2}.$$

σ_{h_i} was calculated during the instrument localization process, as discussed in the paper. The instrument location was solved by using two-way travel times of pings transmitted between the ship-board transducer and the acoustic release attached to the instrument. After solving for the instrument position which minimized the error between expected and measured two-way travel time from various ship locations, the standard deviation of this error is used to estimate σ_{h_i}.

σ_{ray} was estimated using a ray-tracing model in Bellhop with the full-depth CTD cast collected on deployment. The error in the angle of arrival between the isovelocity model and the ray trace model for the entire range of localization was determined. The worst error from ray bending is on the hydrophone pairs that are closest to horizontal, so by estimating the error on a presumed horizontal pair, we overestimate the actual error. For a horizontal pair of hydrophones, the z component of the direction of arrival is related to the incident angle through $s_z = \cos\theta$. Using Eq 1, this means the error added to s due to error in incident angle is

$$\sigma_{\text{ray}} = |\sigma_\theta \sin\theta|,$$

where σ_θ is the uncertainty in the arrival angle for a horizontal pair of hydrophones due to the effects of ray bending.

Estimating $\sigma_{H_{k,l}}$ and incorporating σ_c

$\sigma_{H_{k,l}}$ was calculated during inverting for the hydrophone positions as described in the Methods section. σ_{h_i} is the same variable as in the large-aperture case and was calculated during instrument localization. A CTD was mounted on one of the instruments, and σ_c was calculated as the standard deviation of the temperature measured by the CTD.

To incorporate $\sigma_{H_{k,l}}$, we first expand the dot product in the numerator of Eq 5 to $\vec{H}_{k,l} \cdot \vec{s} = H_x s_x + H_y s_y + H_z s_z$, which leads to an uncertainty equation of:

$$\sigma_{\vec{H}\cdot\vec{s}} = \sqrt{\sigma_{H_{k,l}}^2\left(s_x^2 + s_y^2 + s_z^2\right) + \sigma_s^2\left(H_x^2 + H_y^2 + H_z^2\right)}$$

Since $|\vec{s}| = 1$, this simplifies to $\sigma_{\vec{H}\cdot\vec{s}} = \sqrt{\sigma_{H_{i,j}}^2 + \sigma_s^2 |H_{i,j}|^2}$.

Incorporating σ_c yields:

$$\sigma_{\text{TDOA}(k,l)_{\text{calc}}} = \sqrt{\left(\frac{\sigma_{H\cdot s}}{c}\right)^2 + \left(\text{TDOA}(k,l)_{\text{sml,calc}}\right)^2\left(\frac{\sigma_c}{c}\right)^2}.$$

Estimating $\sigma_{\textbf{xcorr}}$

Abakumov, Roeser, and Shapiro derived an approximation for σ_{xcorr} as [1]:

$$\sigma_{\text{xcorr}} \approx \frac{T_d}{2\pi}\frac{1}{\sqrt{\text{SNR}}}, \quad (7)$$

where T_d is the period corresponding to the dominant frequency in the signal and SNR is the signal-to-noise ratio. We can overestimate σ_{xcorr} by using the minimum frequency in our signal (20 kHz, the cut-off frequency of the high-pass filter) and an SNR of 1. This gives a value of $\sigma_{\text{xcorr}} = 7.96 \times 10^{-6}$ s. The minimum precision we can expect due to a sampling rate of 100 kHz is $1/(100 \times 10^{-3}) = 1 \times 10^{-5}$. Thus, the precision of the cross-correlation is limited more by the sampling rate than the signal properties, and a value of $\sigma_{\text{xcorr}} = 1 \times 10^{-5}$ is used here.

Incorporating σ_{xcorr} yields a total small-aperture uncertainty of:

$$\sigma_{\text{sml}} = \sqrt{\left(\frac{\sigma_{H_{k,l}}}{c}\right)^2 + \left(\frac{||H_{k,l}||}{c}\right)^2 \left(\frac{1}{100^2}\sigma_{h_i}^2 + \sigma_{\text{ray}}^2\right) + \left(\frac{\text{TDOA}(k,l)_{\text{calc}}}{c}\right)^2 \sigma_c^2 + \sigma_{\text{xcorr}}^2}. \quad (8)$$

Large-aperture TDOA uncertainty, σ_{lrg}

Sources of uncertainty on the large-aperture TDOAs are:

- σ_{h_i}, σ_{h_j} – uncertainty in the ith and j^{th} instrument locations,
- $\sigma_{\text{travel time}}$ – uncertainty in the travel time between source and receiver introduced by assuming isovelocity rather than a depth-dependent sound speed profile,
- σ_c – uncertainty due to changes in sound speed at the instrument depth over time, and
- σ_{drift} – uncertainty due to clock drift, and
- σ_{xcorr} – uncertainty due to the precision of cross-correlation in determining the TDOA.

Incorporating σ_{h_i} and σ_{h_j}

Uncertainty in hydrophone positions σ_{h_i} and σ_{h_j} are incorporated into the uncertainty on R_i and R_j. Using Eq. 1, the uncertainty would propagate according to:

$$\sigma_{R_i} = \sqrt{\left(\frac{\partial R_i}{\partial h_{i,x}}\right)^2 \sigma_{h_{i,x}}^2 + \left(\frac{\partial R_i}{\partial h_{i,y}}\right)^2 \sigma_{h_{i,y}}^2 + \left(\frac{\partial R_i}{\partial h_{i,z}}\right)^2 \sigma_{h_{i,z}}^2}. \quad (9)$$

Solving for $\frac{\partial R_i}{\partial h_{i,x}}$ gives:

$$\frac{\partial R_i}{\partial h_{i,x}} = \frac{w_x - h_{i,x}}{R_i}.$$

Plugging this back into Eq. 9 gives:

$$\sigma_{R_i} = \sqrt{\left(\frac{w_x - h_{i,x}}{R_i}\right)^2 \sigma_{h_{i,x}}^2 + \left(\frac{w_y - h_{i,y}}{R_i}\right)^2 \sigma_{h_{i,y}}^2 + \left(\frac{w_z - h_{i,z}}{R_i}\right)^2 \sigma_{h_{i,z}}^2}.$$

Since we use only one value for the uncertainty in the hydrophone position (as discussed above), we can further simplify by assuming $\sigma_{h_{i,x}} = \sigma_{h_{i,y}} = \sigma_{h_{i,z}} = \sigma_{h_i}$:

$$\sigma_{R_i} = \sqrt{\frac{(w_x - h_{i,x})^2 + (w_y - h_{i,y})^2 + (w_z - h_{i,z})^2}{R_i}\sigma_{h_i}^2},$$

which, given Eq. 4, simplifies into:

$$\sigma_{R_i} = \sigma_{h_i}, \tag{10}$$

Estimating $\sigma_{\text{travel time}}$ and σ_c

The variance in difference in travel time between the isovelocity assumption (using $c = 1488.4$ m/s) and the travel time estimated by Bellhop (as mentioned above) gave us an estimate of $\sigma_{\text{travel time}}$ and is an added error on $\text{TDOA}_{\text{calc}}$.

σ_c is the same value used for the small-aperture arrays (measured from the CTD mooring). Incorporation σ_c and Eq. 10 using Eq. 2, the uncertainty on the calculated TDOA becomes:

$$\sigma_{\text{TDOA}_{\text{calc}}} = \sqrt{(\text{TDOA}(i,j)_{\text{calc}})^2 \left(\frac{\sigma_{h_i}^2 + \sigma_{h_j}^2}{(R_i - R_j)^2} + \frac{\sigma_c^2}{c^2} \right) + \sigma_{\text{travel time}}^2}.$$

Using Eq. 3, this can be rewritten as:

$$\sigma_{\text{TDOA}_{\text{calc}}} = \sqrt{\frac{\sigma_{h_i}^2 + \sigma_{h_j}^2}{c^2} + (\text{TDOA}(i,j)_{\text{calc}})^2 \frac{\sigma_c^2}{c^2} + \sigma_{\text{travel time}}^2}.$$

Incorporating σ_{drift} and σ_{xcorr}

Clock drift was estimated using the TDOA of pings from an ADCP mooring deployed at the site. The deviation from the expected TDOA gave an estimate of the clock drifts throughout the deployment. The TDOA fluctuated due to side lobes, mooring tilt, and other noise, so to smooth the clock drift estimate a fifth-order polynomial fit was applied to the drift estimate. This polynomial fit was used to correct the TDOAs for drift in the localization process. σ_{drift} was calculated as the standard deviation of the error between the polynomial fit and the measured drift.

The equation for σ_{xcorr} used in the small-aperture TDOAs assumes that each receiver in the array receives the same signal $s(t + \tau_k)$ with different delays τ_k. However, for the large-aperture TDOA, this assumption may not hold due to the directionality of beaked whale pulses and different channel impulse responses between the source and various receivers. To better account for the uncertainty in arrival time, we used the duration of an averaged *Z. cavirostris* pulse as reported by Baumann-Pickering *et al.* [2], $\sigma_{\text{xcorr}} = 585\mu$s.

Both σ_{xcorr} and σ_{drift} are additive uncertainties on $\text{TDOA}_{\text{meas}}$ in Eq. 3, so the total equation for large aperture uncertainty becomes:

$$\sigma_{\text{lrg}} = \sqrt{\frac{\sigma_{h_i}^2 + \sigma_{h_j}^2}{c^2} + (\text{TDOA}(i,j)_{\text{calc}})^2 \frac{\sigma_c^2}{c^2} + \sigma_{\text{travel time}}^2 + \sigma_{\text{drift}}^2 + \sigma_{\text{xcorr}}^2}. \tag{11}$$

Values and discussion

For the deployment used in this paper, we used the values for σ reported in Tables 1 and 2.

Plugging these values into Eq. 8 and 11 gives:

Table 1. Small-aperture uncertainty values, $\sigma_{\mathbf{sml}}$

Variable	Value
σ_{H_1}	0.274 m
σ_{H_2}	0.289 m
σ_{h_1}	7.7 m
σ_{h_2}	7.5 m
σ_{h_3}	9.8 m
σ_{h_4}	3.7 m
σ_{ray}	0.014 m
σ_c	0.135 $\frac{\text{m}}{\text{s}}$
σ_{xcorr}	1×10^{-5} s

Table 2. Large-aperture uncertainty values, $\sigma_{\mathbf{lrg}}$

Variable	Value
σ_{h_i}	(same as small aperture)
σ_c	0.135 $\frac{\text{m}}{\text{s}}$
$\sigma_{\text{travel time}}$	6.5×10^{-3} s
σ_{drift}	5.66×10^{-4} s
σ_{xcorr}	5.85×10^{-4} s

$$\begin{aligned} \sigma_{\text{sml}} &= \sqrt{3.77 \times 10^{-8} + 2.73 \times 10^{-9} + 4.49 \times 10^{-15} + 1 \times 10^{-10}}, \\ \sigma_{\text{lrg}} &= \sqrt{2.51 \times 10^{-4} + 5.35 \times 10^{-9} + 4.23 \times 10^{-5} + 3.20 \times 10^{-7} + 3.42 \times 10^{-7}}. \end{aligned} \tag{12}$$

As expected, some terms in Eq 11 and 8 contribute orders of magnitude more to the overall uncertainty than others. In both equations, instrument location uncertainty dominates. In the second term of Eq 12, $\frac{1}{R^2}\sigma_{h_i}^2 >> \sigma_{\text{ray}}^2$ for small ranges, so the uncertainty in instrument locations dominates this term. At greater ranges, ray bending contributes more uncertainty and hydrophone position error less, but this makes a reasonable approximation for the ranges over which localization is possible. Uncertainty due to drift may make a more significant contribution in situations where drift cannot be solved for from active sources and would need to be considered in other deployments. Typically clocks are synchronized immediately prior to deployment, then upon recovery the drift is measured, and the drift is assumed linear throughout the deployment. Further exploration is needed to determine the best way to estimate the σ_{drift} from linear drift for these cases.

To simplify future deployments, we propose the following equations as approximations of uncertainty:

$$\sigma_{\text{sml}} \approx \sqrt{\left(\frac{\sigma_{H_{k,l}}}{c}\right)^2 + \left(\frac{||H_{k,l}||}{35c}\sigma_{h_i}\right)^2}, \tag{13}$$

$$\sigma_{\text{lrg}} \approx \sqrt{\frac{\sigma_{hi}^2 + \sigma_{hj}^2}{c^2} + \sigma_{\text{drift}}^2}. \tag{14}$$

The values for σ_{h_i} and $\sigma_{H_{k,l}}$ are calculated for every HARP deployment when the instruments are localized, so this approximation greatly simplifies the process of determining uncertainty. In regions with more variations in sound speed with time or depth, more sources near the surface, or other conditions contributing to uncertainty,

these approximations may not hold. However, for localizing deep-diving sources in locations where the sound speed at depth remains relatively stable, these approximations should suffice.

References

1. Abakumov I, Roeser A, Shapiro SA. Arrival-Time Picking Uncertainty: Theoretical Estimations and Their Application to Microseismic Data. GEOPHYSICS. 2020;85(4):U65–U76. doi:10.1190/geo2019-0589.1.

2. Baumann-Pickering S, McDonald MA, Simonis AE, Solsona Berga A, Merkens KPB, Oleson EM, et al. Species-Specific Beaked Whale Echolocation Signals. The Journal of the Acoustical Society of America. 2013;134(3):2293–2301. doi:10.1121/1.4817832.
