# Supplementary material for: Where’s Whaledo: A software toolkit for array localization of animal vocalizations
Source: PLoS Comput Biol. 2024 May 20;20(5):e1011456. doi: 10.1371/journal.pcbi.1011456 (PMC11142720; doi:10.1371/journal.pcbi.1011456)
Supplement: Attachment — Submitted filename: responseToReviewers.docx [file pcbi.1011456.s003.docx]

Responses to feedback on "Where's Whaledo: A software toolkit for array localization of animal vocalizations.”

Editor comments:

1. L 34 . extra parenthesis.
   RESPONSE: removed parenthesis
2. L. 55. Did you mean “both small-aperture DOA estimates and large-aperture TDOAs”? they are both TDOA estimates at first no?
   RESPONSE: Yes, they are both initially TDOA estimates. Text has been altered to: “Localization can be performed by combining both small-aperture and large-aperture TDOAs”
3. Equation 1. Don’t you need a - in the equation to calculate the difference in arrival times?
   RESPONSE: Yes, this is a typo. It has been corrected.
4. Need to cite Map data
   RESPONSE: included citation on Fig. 6 to GMRT where bathymetry data was obtained.

Reviewer feedback:

1. Has this tool been tested on data from instruments other than HARPs? If not, what considerations should be made? While this seems to be a great tool for 4-channel HARP data, showing it is easily adaptable to other datasets from different tetrahedral arrays would make it more attractive.

RESPONSE: Added paragraph to Discussion (lines 490-496) mentioning that it has only been tested on HARP data and that some adaptations may be required for other datasets. The paragraph states:

*“Where's Whaledo* was developed for and tested specifically on deployments with two 4-channel HARPs and a varying number of single-channel instruments. With some adaptations, *Where's Whaledo* could prove useful with varying instrument configurations, such as large-aperture only or linear arrays. As of publication, the detector and TDOA estimator included on the *Where's Whaledo* GitHub page were developed for Cuvier's Beaked Whales, but the software could be expanded to be used for localizing other sources, such as baleen whales or anthropogenic sounds.“

2. The are few mentions of the directionality issue in the manuscript. First, it should be mentioned that the receivers are omnidirectional (I believe they are?). Second, based on the directionality of beaked whale clicks, I assume you can mostly track whales swimming towards the array vs. whales swimming away from the array. If so, this should be mentioned at least in the discussion.

RESPONSE: Added “omnidirectional” to description of HARPs in Methods / Case Study – Tanner Basin section (lines 313 and 315). Added more to the Discussion about biases potentially introduced due to click directionality. Paragraph (lines 485-489) now reads:

“Click directionality also greatly influences the spatial distribution of tracks obtained, since an encounter is far more likely to be tracked when the animal is facing multiple instruments. This may introduce bias into the types of track obtained at a given site, and a thorough analysis of this spatial bias should be performed for each deployment.”

3. Many localization techniques for odontocetes use multipath. While it is my understanding you don't use these at all, this approach should be mentioned in the literature review, and your discussion could also talk about how using multipath could help improve your current approach. Especially to fill gaps where an animal is not detected on enough instruments to localize using TDOA from various sensors.

RESPONSE: Added a paragraph in introduction mentioning some multipath localization methods and citations. Paragraph, now beginning on line 47, reads:

“Reflections off the surface or refractions due to ray bending, called “multipath arrivals”, can be used in localization [14, 28, 35–39]. Often, multpaths can be used to improve localizations or estimate the range between a source and an array [14, 38]. In cases where accurate models of multipath propagation can be made and there is significant azimuthal variation on these propagation patterns, the measured times of arrival of each multipath can be matched to models to estimate source locations in 3 dimensions from a single hydrophone [28, 37].”

4. It would be nice in Figure 7 / Example 2 to show the TDOA tracks and how you are able to separate the six tracks and associate them to the same source across the different sensors. This example seems to be the most challenging and it would be nice to see in more detail how it is done.

RESPONSE: Added the labeled DOAs to Figure 8 (previously Figure 7). Including the TDOAs would be six additional subplots to the figure, so I’ve only added the DOAs. If the reviewer feels it is necessary, I can either provide it as a supplementary figure or separate Figure 8 into two figures.

5. In the abstract and conclusion you mention how tracking can be useful for things like population density, behavior etc. It would be nice to have a few sentences in the discussion explaining how. Or even better use your results to point out specific things that would be useful for population density, behavior etc.

The following paragraph in the introduction gives a brief overview of how tracking can help with population density estimation:

“For deep-divers, PAM is emerging as an essential method for studying their population structure and dynamics \cite{marques2013estimatingDensity, hildebrandPassiveAcousticMonitoring2015a,solsona-berga2022}. This requires *a priori* knowledge of a number of features, like group size, vocalization rates, and acoustic detection ranges and probabilities. While some studies have estimated these parameters using acoustic models or information known about closely related species or populations, obtaining direct measurements for a specific species and site would likely improve the estimates \cite{marques2013estimatingDensity}. Most of these features can be estimated by reconstructing tracks from acoustic data. Group sizes can be estimated by identifying the number of individual tracks in an encounter. Detection ranges and probabilities can be estimated based on the positions of detected animals.”

In subsequent papers we will be performing more thorough analysis of these features, but since the purpose of this paper is to describe the algorithm, this sort of analysis was omitted.

Responses to comments on the PDF:

REVIEWER: Line 90: says “up to five individuals”, where abstract says “up to six”.
RESPONSE: Corrected so both say “up to five”.

REVIEWER: Fig. 3 is hard to follow …
RESPONSE: Added labels and axes to the figures and regrouped them to make them more clear. Added more text to the description, including a more detailed breakdown of each step in the brushDOA interface.

REVIEWER: line 275: A figure would be helpful to better visualize this method…
RESPONSE: Added a figure (now Figure 5) showing the vectors and their intersection at the source. The figure also shows the variables used to help clarify the equations in this section.

REVIEWER: line 292: I don’t understand this part, particularly the point about redundancy.
RESPONSE: The use of the term “redundancy” and some of the text seemed to muddle the intended meaning of this paragraph. I rewrote the text (lines 299-307) as follows:

“The confidence intervals for this method of localization were obtained using the jackknife variance estimator \cite{shao1989jackknife}. One TDOA and its associated receiver pair is removed from the DOA estimation, and a new DOA is estimated using the remaining five TDOAs. A new whale location is estimated using the intersection point of the newly obtained DOA and the DOA of the other array using Eq. \ref{eq:DOAintersect}. This is repeated for each receiver pair, removing one TDOA and localizing with the remaining 11, until 12 different whale location estimates have been produced. The variance of these location estimates is determined and used in the inverse Student's T distribution to estimate the 95\% confidence intervals.”

REVIEWER: Line 316: Mention that at these short ranges a constant sound speed is usually a good assumption (ideally you’ve even done some very basic modeling/traveltime calculations to convince yourself, but at these range constant sound speed feels safe).
RESPONSE: Added this to the text. Paragraph (lines 324-325) now reads:

“The speed of sound in water depends on various oceanographic conditions, such as temperature, pressure, and salinity, resulting in both temporal and spatial variation in sound speed \cite{mackenzieNineTermEquation1981, chenSpeedSoundSeawater1977, dushawEquationsSpeedSound1992}. However, to simplify computation, a constant sound speed was used for our case study. This approximation is generally acceptable at close ranges. To quantify the error introduced by the constant sound speed approximation, we estimated the variations in sound speed using a CTD (Conductivity, Temperature, Depth) profiler mounted near the instruments at SOCAL_E. Empirical relationships between sound speed, temperature, salinity, and depth were used to estimate sound speed from the CTD measurements \cite{mackenzieNineTermEquation1981, chenSpeedSoundSeawater1977, dushawEquationsSpeedSound1992}. The uncertainty in the assumed sound speed is accounted for in the overall uncertainty of the localization estimates.”

REVIEWER: Line 343: Mention here that the ADCP pings every minute
RESPONSE: Added, “The ADCP pinged approximately every minute at 75 kHz.” (line 368)

REVIEWER: line 396 (“…leading to larger depth confidence intervals …”): Can you explain why this is the case?
RESPONSE: Changed the text to read (line 407-409): “Since errors in DOA angle estimates scale with range, this leads to larger confidence intervals when compared to the much closer second whale.”

REVIEWER: line 412: I assume the clicks from the two species overlap in frequency, causing false detections? Mention the frequency overlap to illustrate the performance of this approach.
RESPONSE: Changed the text to read (lines 421-422), “The overlap in the frequencies of both dolphin and Cuvier's clicks led to a high number of false detections.”

REVIEWER: Fig 7: It would be nice to see the TDOA plots/tracks ….
RESPONSE: same feedback as previously mentioned. Added the DOA plots to the figure and can have the TDOA plots in supplemental if needed.

ADDITIONAL CHANGES

Some additional inconsequential changes were made to help with readability. Most are highlighted on the document.

I changed all references to “Cuvier’s beaked whale” to “*Zc*”. The Society for Marine Mammalogy may soon be changing the name of the animal to avoid using the name of a problematic historical figure, and using the scientific name avoided the problem of having this publication using an outdated name.
